# Enzymatic Fuel Cells: Towards Self-Powered Implantable and Wearable Diagnostics

**DOI:** 10.3390/bios8010011

**Published:** 2018-01-29

**Authors:** Carla Gonzalez-Solino, Mirella Di Lorenzo

**Affiliations:** Department of Chemical Engineering, University of Bath, Bath BA2 7AY, UK; C.Gonzalez.Solino@bath.ac.uk

**Keywords:** self-powered sensors, enzymatic fuel cells, wearable and implantable electronics

## Abstract

With the rapid progress in nanotechnology and microengineering, point-of-care and personalised healthcare, based on wearable and implantable diagnostics, is becoming a reality. Enzymatic fuel cells (EFCs) hold great potential as a sustainable means to power such devices by using physiological fluids as the fuel. This review summarises the fundamental operation of EFCs and discusses the most recent advances for their use as implantable and wearable self-powered sensors.

## 1. The Burden of Chronic Diseases

Worldwide, millions suffer from serious chronic diseases, such as diabetes, asthma or cardiovascular conditions [1]. Inappropriate management of these conditions can lead to constant relapses, hospitalisations and an increased risk of premature death. Currently, chronic diseases cause more than 60% of all annual worldwide deaths, which, considering ageing and the rapid growth in the world’s population, are predicted to increase up to 52 million by 2030 [1]. Chronic diseases are also associated with high healthcare costs. In England alone, the current annual expenditure on chronic care accounts for more than £44 billion, and it is based on obsolete diagnostics techniques that cannot provide real time and rapid monitoring [2].

The development of innovative diagnostic solutions for the effective management of long-term diseases has, therefore, become a necessity to help prevent and/or minimise any associated complications. Point-of-care technologies can allow effective health monitoring, while minimising stress and discomfort to the patients, and therefore enhancing their quality of life, as well as reducing healthcare costs [3]. These devices can also be coupled with wireless transmission systems for rapid and remote data processing, thus heading to the new era of telehealth [4].

## 2. Enzymatic Fuel Cells for Biosensing Applications

Biosensors offer simple, real-time, and direct measurements of analytes in physiological fluids. Detection and monitoring is performed by coupling a biological reaction to a transducer that converts the recognition event into a measurable signal [5]. Although a wide range of transduction methods have been proposed; electrochemical methods are currently the most popular approach, due to their simplicity, easy miniaturisation, robustness and low-cost [6].

Most biosensors require a power source to function, which is usually provided by lithium batteries. Such batteries are, however, difficult to miniaturise, have a limited lifetime from a few months up to several years, and/or require frequent charging, depending on the energy requirements of the device they power [7]. Moreover, these batteries are made of metals of limited availability, and are non-recyclable, thus raising environmental concerns. As such, research into autonomous devices, that also have a smaller environmental footprint, is paramount in future development of point-of-care technologies. In this context, enzymatic fuel cells (EFCs) hold great potential for autonomous biosensing. EFCs are electrochemical devices that exploit the use of redox enzymes to harvest electrical energy from the chemical energy stored in biomolecules (the fuel), and any fluctuations are directly translated into changes in the output current. Consequently, EFCs can work as self-powered amperometric sensors for the target biomolecule (also defined as a biomarker).

**Figure 1:**
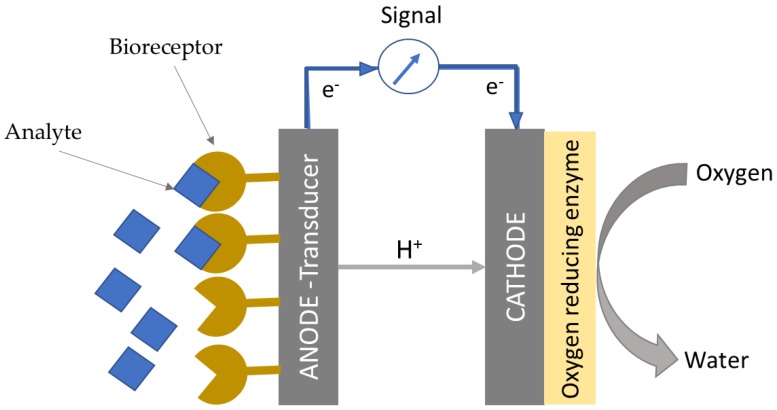
Schematic of an EFC-based biosensor and its detection mechanism. The analyte is detected and oxidised by the bioreceptor, which is usually immobilised at the anode. The electrons released flow across the external circuit to reduce an oxidant, typically oxygen, to water at the cathode.

The system is easy-to-miniaturise, and it is characterised by an extremely simple design, which consists of an anode and a cathode. In particular, the anode acts as the transducer and the redox enzyme at the anode as the bioreceptor (Figure 1). No external transducers are required, as the changes in current directly correlate to the changes in concentration of the target analyte. Contrary to other types of electrochemical sensors, there is no need for a reference electrode, which is prone to failure over time, and, therefore, frequent calibration is not necessary. Considering the simplicity of their design, and the potential for self-powered operation, EFCs represent an exciting avenue for simple, real-time and autonomous point-of-care diagnostics.

**Figure 2:**
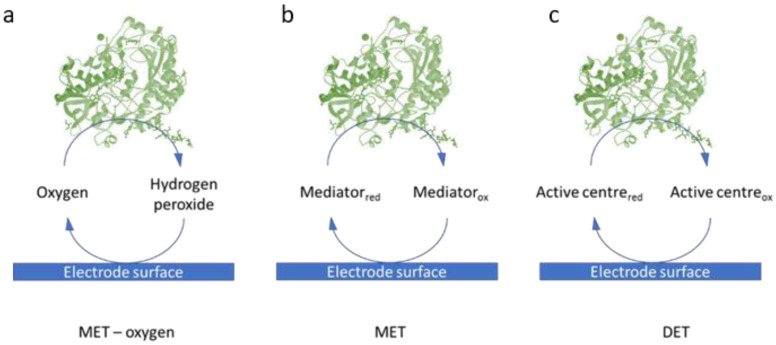
Schematic of the three different electron transfer mechanisms between enzymes and the electrode surface in EFCs: (**a**) Mediated Electron Transfer by oxygen; (**b**) Mediated Electron Transfer by electron shuttles and (**c**) Direct Electron Transfer between the redox centre of the enzyme and the electrode surface.

The sensitivity and overall performance of the EFCs relies on the efficient electron transfer between the enzyme and the electrode surface (Figure 2). In Direct Electron Transfer (DET)-based mechanisms, electrons can be transferred directly from the enzyme to the electrode surface. If the distance is greater than 15 angstroms, however, electron tunneling cannot occur; electron shuttles or mediators are required, leading to a Mediated Electron Transfer (MET) process [8]. Both natural mediators, such as vitamin K_3_ [9] and synthetic mediators, including neutral red [10], methylene green [11], ferricyanide [9] have been employed. The use of mediators, however, introduces a number of challenges, including the risk of leaching from the electrode and poor biocompatibility [12]. Several nanostructures have been also employed to favour the electron transfer rate. These include carbon materials, such as multi-walled carbon nanotubes (MWCNTs) [13], platinum or gold nanoparticles [10], and redox polymers [14]. Oxygen can also act as a mediator, however, challenges such as low concentrations of oxygen and the crossover between anode and cathode, might arise [15].

The mechanism of electron transfer is closely related to the structure of the enzyme employed. Enzymes with deeply buried redox centres, such as glucose oxidase (GOx), must be aided by mediators to connect the enzyme’s redox centre to the electrode and/or overcome their distance [16]. The several strategies implemented include the use of molecular wires and artificial cofactor derivatives [9,14]. A limitation of GOx is that it can use oxygen as the electron acceptor. The high potential required to oxidise oxygen introduces possible interferences with other molecules [17]. Oxygen oxidation can also affect the performance of the oxygen-reducing biocathode [18,19].

The challenges and complications of relying on electron shuttles in MET, however, motivates researchers to explore enzymes capable of DET, and, as such, to find suitable alternatives to GOx. Cellobiose dehydrogenase, an haem enzyme, shows promising DET performance in glucose/oxygen EFCs [20]. This enzyme, however, requires proper engineering to enhance its sensitivity towards glucose and to minimise interferences from other sugars, such as lactose or maltose [21]. Glucose dehydrogenases (GDH) represent an interesting alternative to glucose oxidases [18]. This class of enzymes is unable to use oxygen as the electron acceptor, and instead transfers electrons to natural redox cofactors, such as nicotine adenine dinucleotide (NAD), pyrroloquinoline quinone (PQQ) and flavin adenine dinucleotide (FAD) [17,22]. Among these enzymes, FAD-GDH shows lower redox potential, thus allowing higher power outputs [22]. Contrary to other cofactors, FAD is more tightly bound to the enzyme, which helps prevent its dissociation over time and, consequently, improve the enzyme lifetime [23]. DET by FAD-GDH and its use in EFCs has been successfully demonstrated by Lee et al. [17].

Table 1 summarises the enzymes most commonly used at the anode of EFCs, along with the electron transfer mechanism they require.

Despite the benefits of a DET mechanism, current densities are lower than in a MET if the distance between the electrode and the redox centre is greater than 15 A [24]. Nanostructured electrodes can help overcome this limitation by improving the electrical contact between enzyme and electrode.

The biocathode of EFCs is usually functionalised with enzymes able to reduce oxygen to water, such as laccase [30] or bilirubin oxidase [9] that show DET.

Several studies refer to systems with an abiotic cathode, which is usually based on platinum [31,32] or activated carbon [33]. This approach ensures that the reactions at the anode are the limiting step in the process, with consequent benefits on the sensing performance of the device. These types of systems, however, are referred to as hybrid, since, strictly speaking, they cannot be considered EFCs. The use of a biocathode opens interesting prespectives, as the biocathode itself could act as the biosensing element. The possibility of using cathodic enzymes for biosensing widens the range of possible biomarkers that can be detected such as bilirubin, opening new and exciting biosensing applications [34,35]. Several biocathodes have already been designed to detect toxicants in water, such as arsenic [36] and mercury [37].

## 3. Implantable Enzymatic Fuel Cells

The first EFC implanted in a living organism was reported in 2010 by Cinquin et al. [38]. The EFC consisted of an anode modified with glucose oxidase, and a cathode modified with polyphenol oxidase. Both enzymes were mechanically confined in the electrodes and protected by dialysis bags. The resulting EFC was implanted in the retroperitoneal space of a rat, and generated a power output of 6.5 µW at 0.13 V and an open circuit voltage (OCV) of 0.275 V. Although not operated continuously, this EFC generated a stable power inside the rat for three months without signs of inflammatory reactions. While the results confirmed the exciting possibility of harvesting energy from physiological fluids, the use of dialysis bags and membranes poses several limitations in practical applications.

Subsequent work has focused on the use of nanostructured materials and improved immobilisation methods to enhance power output and stability of EFCs. Particularly, nanostructured materials can improve the electron transfer by shorten the distance between the deeply buried redox centre and the electrode surface [39]. Also, nanostructured materials can be modified to be more biocompatible to improve the stability of enzymes. Carbon has been widely employed as electrode material, due to its good biocompatibility, high electrical conductivity, and high number of docking sites [39]. Different carbon materials have been in particular explored, such as carbon nanotubes, graphene [40] and pressed carbon nanotubes in buckypaper [41]. Katz et al. reported an EFC based on buckypaper modified with 1-pyrenebutanoic acid succinimidyl ester (PBSE), which was functionalised with catalase and PQQ-GDH for the cathode and anode respectively [13]. Both bioelectrodes were implanted in the hemolymph of a snail and connected through an external circuit. Both PQQ-GDH and catalase showed DET, and the implanted EFC generated 7.45 µW over two weeks. These bioelectrodes were also tested by the same authors in clams [41] and lobsters [42].

Later in 2013, Sales et al. developed an EFC based on flexible carbon fibre electrodes for implantation in a rat [10]. The anode was functionalised with neutral red and GOx crosslinked with glutaraldehyde. The cathode was functionalised with dendrimers and platinum nanoparticles. The resulting EFC was implanted inside a polyethylene catheter in the jugular vein of a rat, yielding a power output of 95 µW cm^−2^ at a potential of 0.08 V for 24 h. To date, this EFC is the most promising example of an implanted EFC in a living organism. Its great performance is attributed to the flexible carbon fibre electrodes implemented, which facilitated the implantation in the catheter while enhancing the electron transfer rate.

Osmium polymers have also been widely explored in EFCs, due to their fast electron transfer rates and tunable redox potential [14,43]. Cadet et al. developed a glucose/oxygen fuel cell based on an osmium polymer, functionalised with glucose dehydrogenase, in the case of the anode, and bilirubin oxidase, in the case of the cathode [44]. The resultant EFC achieved a power output of 95 ± 8 µW cm^−2^ at 0.38 V in blood samples containing 5.6 mM of glucose. A membrane was, however, required to prevent biofouling of the electrodes. Despite its excellent electrochemical properties, osmium is a toxic compound [43]. As such, the risk of osmium complexes leaching from the electrode surface raises serious concerns about its use for implantable and wearable devices.

Pankratov et al. developed a glucose/oxygen EFC based on graphite modified with cellobiose dehydrogenase and bilirubin oxidase for the anode and cathode respectively [45]. The EFC was tested in human blood in flow-through mode during 10 minutes by continuously drawing blood from a human subject. In these conditions, the fuel cell generated enough energy to power a low voltage display.

**Figure 3:**
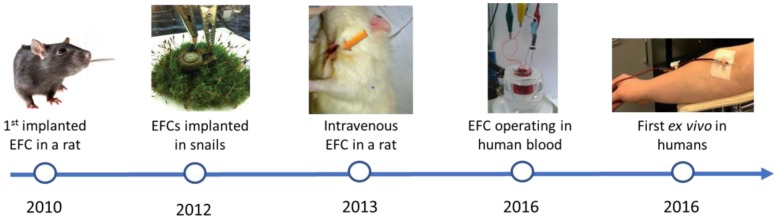
Progression of glucose/oxygen EFC towards implantable applications tested in different animals from references [10,13,38,44,45] with permissions from the Royal Society of Chemistry, American Chemical Society and Elsevier.

Figure 3 outlines the major successes in the research into implantable EFC since the first implanted EFC reported by Cinquin et al. [38]. On the other hand, Table 2 summarises the characteristics of the principal implanted EFCs reported so far. As shown in Table 2, the highest power outputs were obtained with redox polymer and carbon nanostructures, due to the improved electron transfer they are associated with.

Despite the encouraging progress in recent years, practical applications of implantable EFCs are still limited by several challenges. One of the major limitations is undoubtedly the stability of the enzymes. The longest operation time of EFCs in vivo reported so far is three months [38]. Although this represents a great achievement, it is not a long enough time for an implanted device, considering that the average lifetime of a lithium battery-based implanted device is at least five years [7].

Another challenge is posed by the complexity of the blood matrix, which includes a wide range of cells and biomolecules that can precipitate onto the electrode surface and interfere with the electron transfer and/or inhibit the enzymes employed. Possible strategies to prevent or minimise biofouling from interfering compounds, would be the entrapment into membranes of the electrodes, although this would introduce mass transfer limitations. Nafion™, a sulfonated tetrafluoroethylene co-polymer, is commonly used to protect enzyme electrodes in EFCs and enhance the selectivity of the sensor by electrostatic repulsion of unwanted species [48]. Hydrogels, flexible polymeric networks able to absorb water, have also been explored as a means to improve the sensor functionality and stability over time by limiting the diffusion and deposition of molecules in the electrode. Hickey et al. for example, reported the development of a polymeric hydrogel lactate oxidase bioanode based on linear polyethyleneimine (LPEI) modified with dimethyl ferrocene [27].

One of the major concerns of in vivo monitoring is the host immune response after the implantation process and the subsequent long-term interaction with proteins and cells. The human body reacts to unfamiliar materials through a series of reactions that would lead to the formation of a capsule of collagen around the device, which would limit the access of analytes [46]. Moreover, extracellular matrix proteases present in tissues could infiltrate the EFC and degrade the immobilised enzymes [47].

## 4. Wearable Enzymatic Fuel Cells 

**Figure 4:**
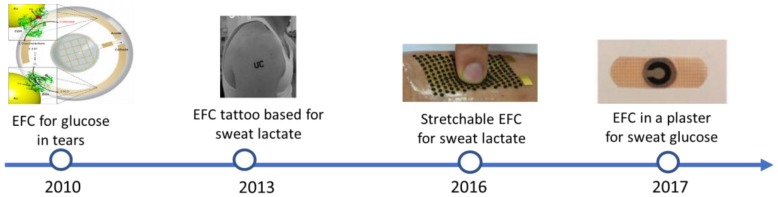
Timeline and progress on wearable EFCs, from the first proof-of-concept in tears to EFCs embedded in plasters from [33,53,54,55] with permissions from the Royal Society of Chemistry, Wiley & Sons and Elsevier.

The challenges associated with implantable EFCs has directed the research towards wearable rather than implantable applications, and, consequently, to the exploration of physiological fluids as an alternative to blood, such as saliva, transdermal fluid, sweat, tears, and urine. These fluids are readily available and do not require invasive implantations or blood draws for in vitro testing. One of the major challenges associated with the use of such fluids, is, however, the lower concentration of target analytes with respect to blood. For example, the concentration of glucose in saliva and tears is up to 20-fold lower than in blood [51,52]. Figure 4 shows the progress in wearable EFC achieved so far.

Extensive research has been devoted to demonstrating the possibility of using EFCs with physiological fluids as an alternative to blood. Falk et al. reported a mediator-less and membrane-less EFC for energy harvesting from glucose in tears [53]. This EFC, based on nanostructured gold microelectrodes coated with cellobiose dehydrogenase for the anode and bilirubin oxidase for the cathode, was characterised by an OCV of 0.57 V and generated a power density of 1 µW cm^−2^, with an operational half-life of two weeks. This research group also demonstrated the possibility of harvesting energy from saliva and sweat using the same electrodes [56].

In 2014, du Toit et al. reported power generation from transdermal extracts from pig skin, obtained by iontophoresis [4]. The EFC was based on highly porous gold functionalised with glucose oxidase and laccase [4]. Cho et al. reported a paper EFC to monitor glucose levels in sweat [33]. The paper was modified with a mixture of two polymers (poly(3,4-ethylenedioxythiophene) and polystyrene sulfonate (PEDOT:PSS). The anode was functionalised with graphene nanoparticles and a chitosan-glucose oxidase complex. The cathode consisted of activated carbon and nickel. In vitro tests showed a good sensitivity to glucose, within the dynamic range of 0.02–1.0 mg glucose mL^−1^. The EFC was tested in human subjects during exercise, however, due to the low production of sweat, continuous monitoring was not possible.

The selection of materials appropriate for wearable applications is a key aspect in this type of EFC, and, as such, has been the focus of a number of studies recently published. Wearable devices should be stretchable, compact, biocompatible, and thin to adapt and integrate to the human body, as well as resilient to wear and tear [55]. Several materials have been tested, as detailed in Table 3.

Synthetic polymers have also been employed to develop microfluidic EFCs [57,58]. For this purpose, a flexible polymer, such as polydimethylsiloxane (PDMS) has been employed [55,59,60]. In more recent years, printed circuit boards (PCB) have been suggested for the development of wearable and affordable diagnostics tools, due to its easy and cheap manufacture, leading to so-called lab-on-a-PCB [61]. PCB is made of different layers of polymer with printed circuits of copper with good mechanical, electrical and thermal characteristics. Although they are generally rigid, flexible PCB can also be manufactured with polyimide sheets [61]. Nonetheless, PCBs have not been explored yet for EFCs.

Paper is an attractive material for the development of affordable and disposable EFCs. As an example, a flexible EFC, based on paper filter modified with cellulose and MWCNTs, has been recently reported. The paper EFC showed an OCV of 0.61 V and a power density of 4.31 µW [62]. One advantage of paper-based devices is that there is no need to integrate micro pumps or micro valves, because the hydrophilic nature of paper itself allows fluids to move by capillary flow. Different techniques have also been reported to develop microfluidic patterns onto paper surfaces. These include wax printing, inkjet printing and screen printing [63]. Fischer et al. for example, reported a paper-based EFC, obtained by screen printing graphite ink electrodes and wax printing microfluidic structures [64]. The resultant EFC showed, however, high internal resistance, which seems to be common in paper-based biological fuel cells [65].

Buckypaper can also allow the development of cheap, light weight, disposable and flexible EFCs [62]. It consists of multiwalled-carbon nanotubes (MWCNTs) compressed into a laminated sheet that still conserves all the properties of CNTs, such as porosity, conductivity, high surface area and low resistivity [27]. Buckypaper also preserves the ability of being easily functionalised, but most importantly is ductile and can be adapted to different surfaces, which makes it ideal for wearable devices. For instance, Gonzalez-Guerrero et al. developed a carbon paper-based EFC for glucose monitoring where the paper was modified with osmium polymers to enhance the electron transfer rate [26]. The resultant EFC presented a linear range up to 15 mM, which is within the desired range for medical monitoring. The reproducibility was, however, poor [26].

**Figure 5:**
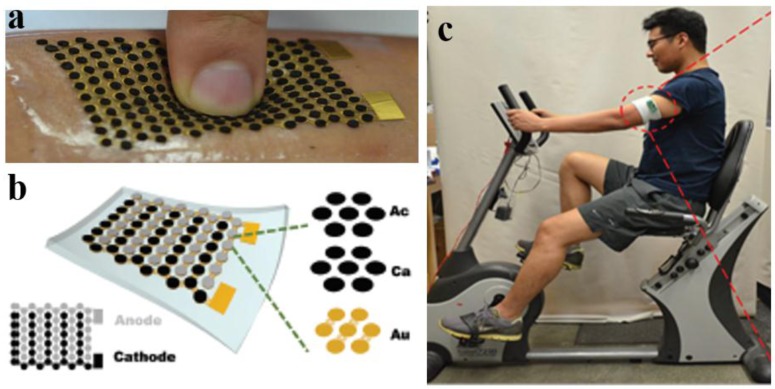
Stretchable EFC for the detection of sweat lactate adapted from reference [55] with permission from the Royal Society of Chemistry: (**a**) schematic of the design of the EFC based on stretchable polymers showing the different layers made of gold (Au), carbon (Ca) and a recognition element in the bioanode/biocathode (Ac) (**b**) prototype EFC attached to human skin and (**c**) EFC monitoring sweat lactate production during exercise.

Bandodkar et al. reported an innovative and flexible EFC fabricated with stretchable materials to allow full integration with the human skin (Figure 5a) [55]. The bioanode and biocathode were divided into islands, interconnected by serpentine bridges, which were free to unwind and deform under stress). This EFC was aimed at detecting and harvesting energy from the lactate present in sweat. The bioanode was modified with LOx immobilised onto a 3D carbon structure and the biocathode was functionalised with Ag_2_O nanoparticles (Figure 5b). The resultant EFC generated a power output of 1 mW cm^−2^, the highest obtained so far with an EFC, which, with the aid of a DC-DC converter, could power a LED when worn by a person while exercising (Figure 5c).

## 5. Self-Powered Detection of Interesting Biomarkers with EFC-Based Sensors 

EFCs hold great potential as self-powered, real-time and in vivo monitoring of a number of biomarkers of interest. So far, research has been focused merely on glucose [66]. Biomarkers, such as lactate and cholesterol, have also been considered [55,67]. Furthermore, by implementing the appropriate enzymes, the use of EFCs could be extended to the detection of: other endogenous molecules, such as neurotransmitters; other metabolites rather than glucose, such as lactate, creatinine or uric acid; and drugs. Table 4 reports biomarkers and analytes of interest, along with the redox enzyme that can be used in an EFC for its self-powered detection. Follows a description of biomarkers/analytes of interest, along with progress on their enzymatic electrochemical detection.

### 5.1. Lactate

Lactate is a product of anaerobic glucose metabolism and plays an important role in maintaining cellular and tissue homeostasis. Abnormal values of blood lactate are related to severe illnesses such as pneumonia, cardiovascular diseases or diabetic coma. Recently, it has been reported that abnormal levels of lactate in blood can be correlated to multiple sclerosis [68]. Finally, lactate monitoring is also of interest to athletes, since high lactate levels are associated with high muscular exertion during anaerobic exercise.

**Figure 6:**
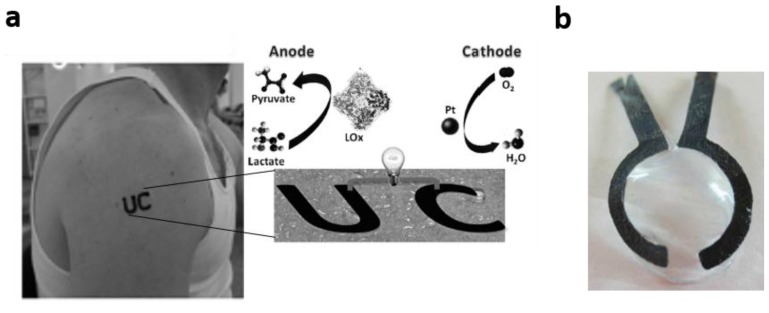
Examples of wearable EFCs for the detection of lactate. (**a**) EFC tattoo sensor for lactate, adapted from [54] with permission from Wiley & Sons; (**b**) EFC contact lens sensor from [11] with permission from Elsevier.

Lactate can be found in physiological fluids such as saliva, tears or sweat, with good correlation to blood lactate levels [69,70,71]. On this premise, Hickey et al. reported an EFC-based sensor for lactate [27]. The enzyme chosen for the anode was lactate oxidase crosslinked with dimethyl ferrocene-modified polymers, whereas the biocathode consisted on carbon felt coated with anthracene modified MWCNTs and bilirubin oxidase. The resultant EFC showed an OCV of 0.57 ± 0.01 V and a power density of 122 ± 5 µW cm^−2^. The lactate detection range was 0–5 mM, which, although too high for physiological values in sweat, is within the concentration range found in tears or saliva [70,71]. Further research in Minteer’s group integrated a lactate/oxygen EFC in a contact lens (Figure 6b) [11].

In 2013, an EFC based on a screen-printed tattoo for lactate monitoring in sweat was reported [54]. The EFC tattoo showed exceptional adaptability to the skin and a good mechanical strength sensor, with no signs of inflammation or irritation to the skin (Figure 6a). The EFC tattoo showed good sensitivity to lactate and a clear distinction in current output when tested on subjects with diverse fitness levels. This tattoo-based self-powered sensor was characterized by a considerable half-life of four weeks and could be easily replaced afterwards. Tattoo-based EFCs present an excellent opportunity for skin-worn biosensors, allowing fully integration in the body.

### 5.2. Cholesterol

Cholesterol is an important biomarker associated with heart and cardiovascular diseases. Normal concentration of total cholesterol in blood should be less than 5.17 mM, although the value can fluctuate with age, weight and gender [67]. Subjects with levels of cholesterol above 8 mM are more prone to suffer from atherosclerosis and associated cardiovascular diseases [72]. Effective real-time monitoring could help to control the levels of cholesterol and reduce the risk of cardiovascular diseases [73]. Sekretaryova et al. reported a membrane-less EFC biosensor for cholesterol, which implemented cholesterol oxidase and Prussian blue [67]. This novel biosensor showed high specificity to cholesterol 26 mA mM^−1^ cm^−2^, with a dynamic range of 0.15–4.1 mM. Recently, Minteer et al. developed an EFC cholesterol sensor made of a cholesterol dehydrogenase anode coupled with a bilirubin oxidase cathode [74]. In particular, bilirubin oxidase was entrapped in a dimethyl-ferrocene LPEI polymer and diaphorase was used as a mediator, while the bioanode was prepared by immobilising the enzyme onto anthracene-modified multi-walled carbon nanotubes drop-cast on buckypaper. This biosensor showed a better dynamic range (5–20 µM) and a sensitivity of 60.12 mA mM^−1^ cm^−2^.

### 5.3. Other Biomarkers

#### 5.3.1. Metabolites

Ketone bodies, such as acetone, 3-hydroxybutyrate and acetoacetate, are produced in diabetic ketoacidosis, a serious progression of diabetes. Ketone bodies come from the metabolism of fats when insulin levels are low and glucose cannot get inside the cells [75]. Thus, this complication is usually observed in patients with diabetes type 1, where the production of insulin is reduced or impaired [76]. The normal concentration of ketone bodies is below 50 µM, but it can be higher than 25 mM in serious ketoacidosis [75]. Diabetic ketoacidosis is a life-threatening complication that requires immediate hospitalisation. Thus, along with glucose, monitoring the levels of ketone bodies can help to drastically reduce diabetes-related complications. Ketone bodies can be electrochemically monitored by quantifying the concentration of 3-hydroxybutyrate with the enzyme 3-hydroxybutyrate dehydrogenase (3-HBDH). Recently, an amperometric three-electrode 3-HBDH-based biosensor to monitor ketone bodies in blood samples was developed [77]. The resultant device showed a linear range within concentrations of 3 µM and 0.4 mM, and a limit of detection of 0.1 µM. Yet, no self-powered biosensor for ketone bodies has been reported so far.

Uric acid is an end-product of protein metabolism. High levels of uric acid in blood indicate a dysregulation in the metabolism of purines, which is related to a variety of diseases, such as hyperuricemia, gout, and renal syndrome. The normal concentration of uric acid in blood range from 200 to 400 µM, and between 100 µM and 250 µM in saliva [78,79]. Nevertheless, recent studies proposed the possible electrochemical detection of uric acid in saliva [80].

Creatinine is another by-product of protein metabolism normally cleared in the kidneys and eliminated in urine. A malfunctioning of the kidneys could, however, lead to an accumulation of metabolic waste and increase the levels of creatinine in blood. Normal values of creatinine in blood range between 53 and 115 µM [79]. Moreover, a positive correlation has been found between salivary and blood levels of creatinine [81] where normal salivary levels are about 0.20 mg dL^−1^ increasing up to 2.6 mg dL^−1^ in patients with chronic kidney disease [81,82]. The amperometric detection of creatinine is possible but involves three different enzymes to be immobilised, increasing the complexity of the device: creatinine amidohydrolase, creatine aminohydrolase, and sarcosine oxidase [83].

Sarcosine is associated with Prostate Cancer (PCa), since patients showed increased levels of sarcosine in urine with the cancer progression [84]. In conjunction with other biomarkers, therefore, it can be considered for PCa clinical diagnosis and monitoring [85]. Abnormal concentrations of sarcosine in urine range from 1 to 20 µM thus, extremely sensitive biosensors are required for its detection [86]. The electrochemical detection of sarcosine through the use of the enzyme sarcosine oxidase, has been successfully demonstrated [48]. The work by Rebelo et al. is a very interesting example, based on a three-electrode sensor with a dynamic range between 10 and 100 nM and a limit of detection of 16 nM. No EFC-based biosensors for sarcosine in urine have, however, been reported yet.

#### 5.3.2. Neurotransmitters

In vivo neurochemical sensing is gaining increasing attention as a way to study and understand brain function. Regular monitoring of neurotransmitters could help to improve the life of millions of patients with neurodegenerative disorders, such as Alzheimer’s Disease or Parkinson’s Disease. Acetylcholine (ACh) is an important neurotransmitter involved in cognition, memory and in maintaining the muscle tone. The dysregulation of ACh is correlated with a variety of neurophysiological disorders, such as Parkinson’s Disease, Alzheimer’s Disease, myasthenia gravis or schizophrenia. Although ACh is found in the peripheral and central nervous system, it can be also detected in blood, however, the concentrations are extremely low, normally between 7.6 and 9.7 nM [79]. The release of this neurotransmitter occurs at the millisecond and thus requires rapid and sensitive methods for monitoring and detection. In 2017, Moreira et al. reported the first EFC-based sensor for the continuous monitoring of ACh in blood. This self-powered biosensor was based on highly porous gold electrodes modified with acetylcholine esterase [31]. Self-powered monitoring of neurochemicals would minimise any disturbance to electric activities of the surrounding neurons in the brain. Progress in the field is the subject of a recent review [87].

#### 5.3.3. Drugs

Self-powered biosensors could allow the design of targeted and personalised therapies, via the monitoring of drug uptake and metabolism. An interesting example is given by Levodopa, or L-DOPA, the most common drug for treating Parkinson’s Disease. This drug can cross the blood-brain barrier where it is converted to dopamine [79]. Levodopa is oxidised to *o*-quinones by the redox enzyme tyrosinase, a monophenol mono-oxygenase [88]. Thus, a tyrosinase-based EFC could allow the monitoring of Levodopa levels in blood for personalised drug dosage.

Ethanol detection in physiological fluids is an index of high-risk alcohol consumption. It can be detected in transdermal fluids electrochemically [89]. Recently, Ruff et al. developed an EFC for the detection of ethanol through the use of two different enzymes alcohol oxidase and alcohol dehydrogenase [29]. The resultant self-powered sensor showed a dynamic range of 0.1–1 mM.

## 6. Conclusions and Further Perspectives

Every year, millions of people worldwide are diagnosed with chronic diseases. If not effectively managed, these diseases can degenerate into serious complications, which can eventually lead to death. Enzymatic fuel cells hold great potential as a unique self-powered means for the real-time, rapid and cost-effective monitoring of key biomarkers, associated with the patient’s health. Exciting progress has been made during the past few years, both in the field of implantable and wearable diagnostics, showing the possibility of implementing this technology with a variety of physiological fluids, such as blood, tears, saliva, sweat and transdermal extracts. The full potential of this technology has yet to be explored, considering the variety of biomarkers that can be screened. Current literature is mainly focused on glucose and lactate with just a few studies reported for other biomarkers. The use of alternative enzymes in both anode and cathode can bring the possibility of detecting new biomarkers in a self-powered manner.

Practical applications are, however, hampered by the short lifetime of the enzyme. If not properly overcome, this challenge will particularly limit any further progress in the use of EFCs for implantable devices. On the other hand, lifetimes of months, weeks, and in some cases even days, can be acceptable for wearables, provided that the system is affordable enough. Another challenge is represented by the low baseline current generated, usually due to a poor electrical contact between the redox centre of the enzyme and the electrode, and aggravated by the very low concentrations of analytes in physiological fluids. Low baseline current can limit the sensitivity and can be addressed, for example, by engineering an electrodes array that would scale up the signal generated by a single fuel cell.

Finally, more research needs to be devoted to the integration of the EFC with the human body. Implantable devices must minimise any risk of immune response, while wearable biosensors must be flexible but also mechanically resistant.

## Figures and Tables

**Table 1:** Summary of the most common enzymes employed in EFCs for the bioanode.

Enzyme	Cofactor	Electron Transfer Mechanism	Substrate	Reference
Glucose oxidase (GOx)	FAD	MET	Glucose	[25]
Glucose dehydrogenase (NAD-GDH)	NAD	MET	Glucose	[26]
Glucose dehydrogenase (PQQ-GDH	PQQ	MET	Glucose	[13]
Glucose dehydrogenase (FAD-GDH)	FAD	DET	Glucose	[17]
Lactate oxidase (LOx)	FAD	MET	Lactate	[27]
Lactate dehydrogenase (LDH)	NAD	MET	Lactate	[11]
Cellobiose dehydrogenase (CDH)	Haem	DET	Glucose	[28]
Alcohol dehydrogenase (ADH)	NAD	MET	Alcohol	[29]

**Table 2:** Summary of the implanted and implantable EFCs developed.

Anode	Mediator	Cathode	Implantation Place	Power Density	OCV (V)	Reference
Graphite discs containing glucose oxidase, ubiquinone and catalase	Ubiquinone	Graphite modified with polyphenol oxidase and quinone, surrounded by a cellulose dialysis membrane	Retroperitoneal space in rats	6.5 µW	0.275	[38]
Buckypaper modified with PBSE and glucose dehydrogenase	PQQ	Buckypaper modified with PBSE and laccase	Snail hemocoel	30 µW·cm^−2^	n/a	[13]
FCF microelectrodes modified with neutral red and glucose oxidase crosslinked with glutaraldehyde	Neutral red	FCF modified with PAMAM-G4 dendrimer and PtNPs	Intravenous in a rat	95 µW·cm^−2^	0.125	[10]
Buckypaper modified with PBSE and glucose dehydrogenase	PQQ	Buckypaper modified with PBSE and laccase	Clams visceral mass	40 µW·cm^−2^	0.3–0.4	[41]
Carbon rod modified with osmium polymers and glucose oxidase crosslinked with PEGDGE	Osmium polymers	Bilirubin oxidase crosslinked with osmium polymers using PEGDGE and grafted onto carbon rods	Cockroachabdomen	55 µW·cm^−2^	n/a	[49]
Buckypaper modified with PBSE and glucose dehydrogenase	PQQ	Buckypaper modified with PBSE and laccase	Cremaster tissue in a rat	0.175 µW·cm^−2^	0.140	[50]
Carbon fibre modified with glucose dehydrogenase crosslinked with osmium polymers	Osmium polymers	Carbon fibre modified with bilirubin oxidase crosslinked with osmium polymers	In vitro with human blood	68.1 µW cm^−2^	0.65	[44]
Cellobiose dehydrogenase adsorbed onto graphite electrode	DET	Bilirubin oxidase adsorbed onto graphite electrode	Ex vivo with human blood	0.74 µW	0.31	[45]

PBSE: 1-pyrenebutanoic acid succinimidyl ester; PQQ: pyrroloquinoline-quinone; FCF: flexible carbon fibre; PAMAM-G4: polyamidoamine dendrimer 4th generation; PtNPs: platinum nanoparticles; PEGDGE: polyethyleneglycol diglycidyl ether.

**Table 3:** Summary of wearable EFCs reported so far, with specification on the type of biofluid and material.

Biocatalysts (Anode/Cathode)	Material	Biomarker/Fuel	Biofluid	OCP (V)	Power Output (µW cm^−2^)	Reference
CDH/bilirubin oxidase	Gold wires attached to a contact lens	Glucose	Tears	0.57	1	[53]
Lactate oxidase/laccase	Toray carbon paper	Lactate	Tears	0.64 ± 0.03	61.2 ± 9.2	[57]
LDh-NAD/bilirubin oxidase	Buckypaper	Lactate	Tears	0.413 ± 0.06	8.01 ± 1.4	[11]
LOx/Ag_2_O nanoparticles	Screen printed in strecthable polymer	Lactate	Sweat	n/a	1000	[55]
Gox/activated carbon	Filter paper modified with PEDOT:PSS	Glucose	Sweat	n/a	n/a	[33]
Lactate oxidase/carbon ink modified with platinum black	Screen printed tattoo	Lactate	Sweat	n/a	25	[54]

LDh_NAD: lactate dehydrogenase dependent on nicotinamide dinucleotide; PSS: (poly(3,4-ethylenedioxythiophene) and polystyrene sulfonate copolymer; GOx: glucose oxidase.

**Table 4:** Potential biomarkers and associated diseases that can be detected with an EFC biosensor.

Biomarker/Analyte	Enzymes	Application/Condition	Biofluid	Type of Sensor
Metabolites				
Glucose	Gox/GDH/CDH	Diabetes	Blood, saliva, sweat	Wearable
Lactate	LOx/LDH	Hypoxia	Blood, saliva, sweat	Wearable
Cholesterol	ChOx/ChDH	Atherosclerosis/heart failure	Blood	Implantable
Ketone bodies	3-HBDH	Diabetes	Blood	Implantable
Uric acid	Uricase	Renal syndrome	Blood, saliva	Wearable
Creatinine	SOx	Chronic kidney disease	Blood, saliva	Wearable
Sarcosine	SOx	Prostate Cancer	Blood, urine	Implantable
Bilirubin	BOx	Jaundice/Kernicterus	Blood	Implantable
Neurotransmitters				
Glutamate	GlOx	Neurodegenerative diseases	Brain	Implantable
Acetylcholine	AChE-Choline oxidase	Neurodegenerative diseases	Blood	Implantable
Other analytes				
Levodopa	Tyrosinase	Parkinson treatment	Blood	Implantable
Alcohol	ADH/AOx	Alcohol abuse	Blood	Wearable

GOx: glucose oxidase; GDH: glucose dehydrogenase; CDH: cellobiose dehydrogenase; LOx: lactate oxidase; LDH: lactate dehydrogenase; ChOx: cholesterol oxidase; ChDH: cholesterol oxidase; 3-HBDH: 3-hydroxybutyrate dehydrogenase; SOx: sarcosine oxidase; GlOx: glutamate dehydrogenase; AChE: acetylcholinesterase; ADH: alcohol dehydrogenase; AOx: alcohol oxidase.
